# Editorial: Mineral solubilizing microorganisms (MSM) and their applications in nutrient bioavailability, bioweathering and bioremediation, volume III

**DOI:** 10.3389/fmicb.2025.1662759

**Published:** 2025-08-15

**Authors:** Muhammad Zahid Mumtaz, Maqshoof Ahmad, Adnan Mustafa

**Affiliations:** ^1^Institute of Molecular Biology and Biotechnology, The University of Lahore, Lahore, Pakistan; ^2^College of Agronomy, Gansu Agricultural University, Lanzhou, China; ^3^Department of Soil Science, Institute of Soil and Water Resources, The Islamia University of Bahawalpur, Bahawalpur, Pakistan; ^4^Key Laboratory of Vegetation Restoration and Management of Degraded Ecosystems, South China Botanical Garden, Chinese Academy of Sciences, Guangzhou, China

**Keywords:** phosphate solubilizing microorganisms, potassium solubilizing microorganisms, zinc solubilizing microorganisms, mineral solubilizing microorganisms, mineral-microbe interactions, plant-microbe interactions

## 1 Introduction

Mineral-solubilizing microorganisms (MSM) are key drivers of mineral transformation in soil, and they play a pivotal role in nutrient cycling, environmental detoxification, and geochemical processes. These diverse microbial communities possess remarkable abilities to solubilize and mobilize essential macro- and micronutrients and improve nutrient availability in soil for plant uptake. They drive essential biogeochemical processes by releasing nutrients from insoluble mineral forms. Additionally, MSM are involved in the natural breakdown of rocks and minerals and offer ecologically friendly remediation of the contaminated environment through metal chelation, acidification, and redox transformation. Vol III of our Research Topic series highlights cutting-edge insights into the mechanisms and ecological significance of MSM, exploring their potential applications in sustainable agriculture, soil fertility enhancement, ecosystem restoration, and remediation technologies.

## 2 MSM-mediated nutrient bioavailability

Microorganisms are an integral part of soil biogeochemical cycles involved in promoting soil fertility and the transformation of minerals. MSM have emerged as key microbial agents in agricultural ecosystems due to their ability to transform insoluble minerals into bioavailable forms. They performed extensive functional roles that promote nutrient uptake in plants by catalyzing mineral weathering and the solubilization of minerals. This editorial compiled the research published in Vol III of the Research Topic by discussing various studies that explore diverse interactions between MSM taxa with minerals and plants and drive critical transformations of minerals. The functional role of MSM in enhancing nutrient bioavailability is a rapidly growing area of research in soil microbiology. MSM are involved in the solubilization of silica and silicate minerals, which are gaining attention due to the role of silicon in plant stress tolerance and improving crop productivity. Lei et al. have conducted a bibliometric analysis of global research trends spanning from 1948 to 2024 in the application of phosphate-solubilizing microorganisms, which revealed a rapid growth in this field since 2018. Initially, the research focus was on the application of *Azospirillum brasilense* along with rock phosphate, which shifted toward alleviation of abiotic stresses, especially drought and salt stress, and improvement in crop productivity. This study recommends further exploration of phosphate-solubilizing microorganisms in improving nutrient availability, soil health, and mitigation of abiotic stresses to support sustainable agriculture.

A study by Zhang Y. et al. reports an enhancement in phosphorus-associated *Arthrobacter* sp. M4 and *Sordariomycetes* 2 MS-M4 activity in response to long-term application of swine manure. These microbial species converted available phosphorus into organic phosphorus under high carbon and phosphorus soil conditions through biological immobilization. Meanwhile, they decompose soil organic carbon and promote phosphorus concentration under limited carbon and phosphorus soil conditions by demonstrating their capacity to transform phosphorus. These phosphorus-associated microorganisms significantly promoted phosphorus availability in soil under long-term swine manure with NPK fertilizer. Similarly, Arunachalam et al. demonstrate the potential of plant growth-promoting *Serendipita indica* and vesicular arbuscular mycorrhizae in improving soil fertility, crop yield, and nutrient uptake in onion. They recommend the application of these microorganisms along with recommended chemical fertilizers to promote soil health, onion yield, and bulb quality. Further, Jalal-Ud-Din et al. report zinc solubilization by *Staphylococcus succinus* CLS1, *Priestia aryabhattai* CLS2, and *Priestia megaterium* CLS9 isolated from canola. These strains demonstrate *in vitro* N_2_ fixation, production of indole acetic acid, hydrogen cyanide, exopolysaccharides, and siderophores. These strains promoted plant growth and yield attributes and oil contents in canola.

In a study by Maharjan et al., 24 rhizobacterial strains demonstrated silica solubilization and promoted host maize seedling growth. These strains upregulated antioxidant enzymes, including catalase, superoxide dismutase, peroxidase, polyphenol oxidase, and phenylalanine ammonia-lyase. These potent silica-solubilizing strains belonged to *Enterobacter* sp., *Klebsiella* sp., and *Serratia surfactantfaciens* and were recommended as a potential bioinoculation for the development of silicon-based development of biofertilizer. Luqman et al. applied a consortium of *Bacillus megaterium* ZR19, *Paenibacillus polymyxa* IA7, and *Bacillus* sp. IA16, along with recommended NPK and micronutrients in cotton, and reported the increase in antioxidant enzymes, root and shoot growth, and reproductive and yield traits of cotton under arid climate conditions. These microbial consortia demonstrated biocontrol ability against sooty mold and improved post-harvest soil biological and chemical properties. The synergistic potential of microbial consortia in soybeans under field conditions was studied by Rafique et al.. They inoculated a tripartite combination of *Bradyrhizobium diazoefficiens, Bacillus* sp. MN54 and *Piriformospora indica*, and reported a significant increase in germination rate, plant height, root nodulation, photosynthetic pigments, and leghemoglobin levels. These microbial consortia promoted nitrogen, phosphorus, and micronutrient accumulation in soybean grains and stover. Gato et al. report increasing grain and oil yields for castor beans through a consortium of *Azospirillum brasiliense, Bacillus subtilis*, and *Pseudomonas fluorescens*. This microbial consortium also promoted N uptake in shoots and grains of castor beans. Further, Wang et al. show a shift in microbial and chemical processes involved in weathering dynamics of purple parent rocks. They observe a reduction in pH and an increase in availability of nitrogen and phosphorus by high fertilizer application rate, which impacts weathering through modifying chemical properties and enriching microbial community structure, which ultimately accelerates the breakdown of purple parent rocks.

## 3 MSM-mediated bioweathering

MSM play a pivotal role in mineral bioweathering processes by transforming stable and geochemically resistant minerals into bioavailable forms for plant uptake. Bioweathering is a biologically driven transformation and dissolution of minerals by MSM, which are capable of mobilizing essential nutrients from insoluble mineral forms. This microbial bioweathering process takes place through microbial acidolysis, chelation, enzymatic degradation, and redox reactions that collectively break down primary and secondary mineral structures. Vol III of our Research Topic publishes work on mineral solubilization by MSM, including phosphate-solubilizing microorganisms (Arunachalam et al., Lei et al., Zhang Y. et al.), zinc-solubilizing rhizobacteria (Jalal-Ud-Din et al.), and silica-solubilizing rhizobacteria (Maharjan et al.), which play a pivotal role in natural rock weathering and soil genesis processes. The application of such MSM offers a sustainable biological tool for ecosystem restoration through mineral weathering in degraded geological substrates of nutrient-poor or extremely weathered landscapes. In bioweathering, the action of MSM is not only limited to nutrient solubilization, but these microorganisms also release the elements from polymineralic substrates. These microbial processes are central to nutrient dynamics in agroecosystems, especially under conditions of nutrient depletion or intensive cropping. The diversity and adaptability of MSM across various edaphic and climatic conditions highlight their value as biological agents for increasing soil nutrient cycling through bioweathering.

## 4 MSM-mediated bioremediation

Environmental contaminants, especially heavy metal pollution, continue to pose an ecological threat to both agricultural and mining-impacted lands. Utilization of MSM for bioremediation is a cost-effective and sustainable remediation strategy. MSM indirectly support phytoremediation by extracting, stabilizing, and/or degrading environmental contaminants and promoting plant growth, nutrient acquisition, and stress tolerance. They detoxify soils, improve nutrient availability and plant health, and represent a paradigm shift from traditional remediation to microbe-mediated ecological restoration. Vol III of our Research Topic has published four articles addressing the contamination of lead, cadmium, and arsenic through the application of MSM. A study by Zhang C. et al. showcases insights into the bioremediation of lead and cadmium in phosphate mining wastelands through phosphate-solubilizing *Bacillus cereus* along with biochar. They reported an increase in the phosphorus availability and soil microbial communities, and a decrease in the extractable lead and cadmium concentration. The immobilization of lead and cadmium was caused by the main functional flora of *Janibacter, Lysobacter, Ornithinimicrobium, Bacillus*, and *Salinimicrobium*.

Gul et al. have explored recent advancements in lead remediation strategies through MSM microorganisms. They show the detoxification strategies of lead through biosorption, bioprecipitation, biomineralization, and bioaccumulation, emphasizing how microbes convert toxic lead into non-toxic or less mobile forms. Moreover, advances in genetic engineering have further equipped microbes with resistance traits, enabling their survival and remediation of lead in highly toxic environments. Shahid et al. have extended the cadmium remediation by applying *Klebsiella* strains with jasmonic acid in cauliflower. This biointegrated strategy not only reduces cadmium uptake in cauliflower roots and curds but also enhances plant growth, enzymatic defense mechanisms, and nutrient accumulation. The findings highlight the critical role of plant-microbe synergies in building resilience under metal stress. Similarly, Zaheer et al. explore the co-application of *Azospirillum brasilense* and seaweed extract to combat arsenic toxicity in wheat. This dual treatment improved nutrient availability and plant physiological attributes by reducing arsenic uptake in wheat. Such combinations highlight a promising future in integrating biofertilization and stress mitigation through MSMs and organic stimulants. These studies highlight the possible application of MSM in the alleviation of heavy metal stress and soil restoration. The application of microbial inoculants with soil amendments demonstrated a powerful, environmentally friendly solution to heavy metal contamination. Empowering soils with microbial inoculants is not just a remediation strategy, but it is a sustainable path toward ecological restoration and agricultural sustainability.

